# Identification of miRNAs and Their Target Genes in Peach (*Prunus persica* L.) Using High-Throughput Sequencing and Degradome Analysis

**DOI:** 10.1371/journal.pone.0079090

**Published:** 2013-11-13

**Authors:** Xiaoyan Luo, Zhihong Gao, Ting Shi, Zongming Cheng, Zhen Zhang, Zhaojun Ni

**Affiliations:** College of Horticulture, Nanjing Agricultural University, Nanjing, Jiangsu, People’s Republic of China; Auburn University, United States of America

## Abstract

MicroRNAs play critical roles in various biological and metabolic processes. The function of miRNAs has been widely studied in model plants such as Arabidopsis and rice. However, the number of identified miRNAs and related miRNA targets in peach (*Prunus persica*) is limited. To understand further the relationship between miRNAs and their target genes during tissue development in peach, a small RNA library and three degradome libraries were constructed from three tissues for deep sequencing. We identified 117 conserved miRNAs and 186 novel miRNA candidates in peach by deep sequencing and 19 conserved miRNAs and 13 novel miRNAs were further evaluated for their expression by RT-qPCR. The number of gene targets that were identified for 26 conserved miRNA families and 38 novel miRNA candidates, were 172 and 87, respectively. Some of the identified miRNA targets were abundantly represented as conserved miRNA targets in plant. However, some of them were first identified and showed important roles in peach development. Our study provides information concerning the regulatory network of miRNAs in peach and advances our understanding of miRNA functions during tissue development.

## Introduction

MicroRNAs (miRNAs) are small, endogenous, non-coding small RNAs that negatively control gene expression by cleaving or inhibiting the translation of target gene transcripts [1], [2]. Increasing evidence indicates that miRNAs play critical roles in nutrient homeostasis, developmental processes, abiotic stresses and pathogen responses [3]–[5]. Plant miRNAs are transcribed by RNA polymerase II to generate primary miRNA (pri-miRNA) transcripts and then cleaved to miRNA precursors (pre-miRNAs), catalysed by a Dicer-like enzyme (DCL1) [6], [7]. The pre-miRNA is further cleaved to a miRNA duplex (miRNA:miRNA*) and one of the strands of this duplex is incorporated into the RNA-induced silencing complex (RISC) [8]. The miRNA* strand is usually degraded, although in some cases it also accumulates at a lower level [9]. The mature miRNA strand guides ARGONAUTE (AGO) to complementary target mRNA resulting in silencing of the target gene [10], [11].

Plant miRNAs have been identified mostly by three strategies: traditional Sanger sequencing, computational prediction and high-throughput sequencing technology. Traditional Sanger sequencing has been used for the identification of conserved miRNAs in Arabidopsis [12], [13], rice [14]–[16], wheat [17], moss [18] and poplar [19]. A computation-based approach can predict miRNAs based on high miRNA conservation, but it cannot be characterized and limited to the discovery of false positive [20]. The application of deep sequencing has greatly facilitated small RNA discovery and increasingly more miRNAs, especially species-specific miRNAs have been identified in Arabidopsis [9], [21], soybean [22], Populus [5], [23], cotton [24], Japanese apricot [25] and *Hevea brasiliensis* [26].

Peach (*Prunus persica* L.) is one of the most important fruit crops worldwide and serves as a genome model for fruit trees in the *Rosaceae*, such as apple, cherry and plum [27]. In 2010, the Genome Sequencing Project of the peach double haploid cultivar ‘Lovell’ was completed, which generated ∼230 Mb genome sequence and 202 assembly scaffolds (http://www.rosaceae.org/node/355). Peach has some unique biological facets not commonly found in model organisms such as a 3–5 year juvenility period before the trees flower and fruit, the formation of fleshy fruit with a hardened endocarp and dormancy triggered by cold weather and/or short photoperiod in the autumn [28]. Recently, some groups have predicted or identified miRNAs and their target genes in peach. Zhang et al. [29] and Gao et al. [30] independently identified miRNAs by computational prediction and experimentally by verifying miRNAs via RT-qPCR and two miRNA target genes were validated using RLM-RACE. Several miRNAs responsive to drought [31] or chilling [32] and miRNAs in different tissues [28] were identified by high-throughput sequencing in peach. However, the majority of miRNA targets in peach were predicted by bioinformatic approaches, and only 80 miRNA target genes were identified by high-throughput degradome library sequencing, which has been developed for the global identification of targets of miRNAs in Arabidopsis [33], [34], rice [35], [36], soybean [37] and grapevine [38] and other plants.

No peach miRNA are annotated in the miRBase (version 19.0). To identify more conserved and peach-specific miRNAs and their target genes and to understand further the mechanism of miRNA-regulated target genes during tissue development in peach, a small RNA library and three degradome libraries were constructed from three different tissues for deep sequencing.

## Results

### Small RNA Sequencing Analysis

**Figure 1:**
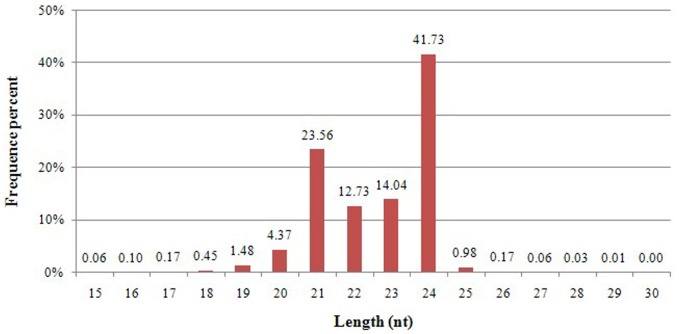
Sequence length distribution of peach sRNAs.

To identify miRNAs involved in tissue development, a small RNA library was constructed from peach from young leaves, young stems and flowers. A total of 14,781,274 high quality reads were obtained from the small RNA library. The small RNA length distribution (15–30 nt) of the library showed that the proportion of 21–24 nt small RNAs reached 92.06%. The 24 nt class was the most abundant (41.73%) group of sRNAs followed by 21 nt sequences (23.56%) (Fig. 1). After removal of adaptor, insert, poly (A) sequences and those reads which were smaller than 18 nt and larger than 30 nt, the remaining 14,693,759 clean reads accounted for 99.41% of the total reads. Clean read sequences were divided into rRNA, tRNA, snRNA, snoRNA, miRNA and unannotated reads, by blasting against Rfam, NCBI and miRBase databases (Table 1). Among the sRNA sequences, unannotated reads accounted for 86.85% of the total (unique, 72.21%), which was the most abundant class.

**Table 1:** Summary of small RNA sequencing data analysis.

Type	Unique reads	Count %	Total reads	Count %
Clean sequence	4,259,405	100	14,693,759	100%
exon_antisense	94,683	2.22	344,177	2.34
exon_sense	132,524	3.11	532,607	3.62
intron_antisense	92,540	2.17	294,133	2.00
intron_sense	129,476	3.04	669,370	4.56
rRNA	63,681	1.50	1,183,987	8.06
tRNA	6,400	0.15	154,868	1.05
snRNA	3,026	0.07	29,905	0.20
snoRNA	666	0.02	2,064	0.01
miRNA	45,430	1.07	871,626	5.93
unannotated	3,690,979	86.65	10,611,022	72.21

### Identification of Conserved miRNAs and Evolutionary Conservation

**Figure 2:**
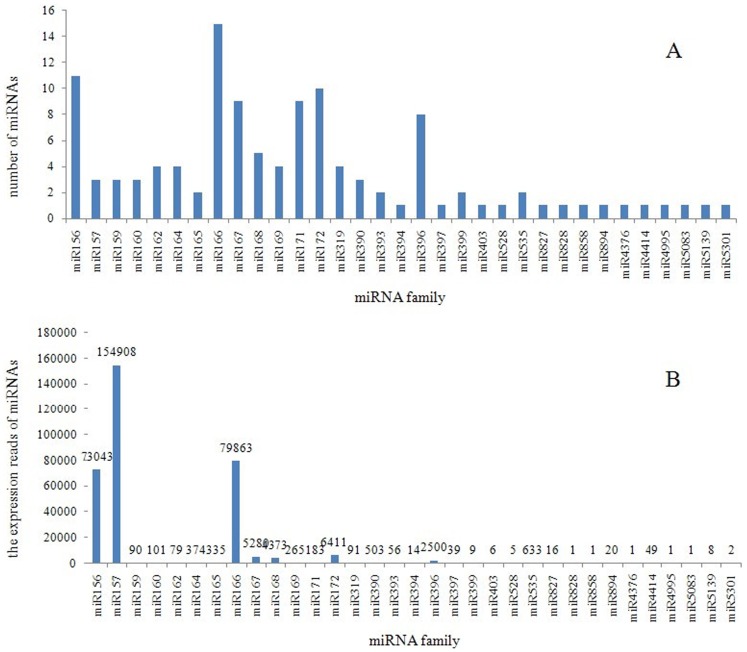
Member numbers of each identified conserved miRNA family in peach (A). The expression read of miRNA families in peach (B).

To identify conserved miRNAs, clean reads of the library were blasted against known plant miRNAs from the miRBase 18.0. Only perfectly matched sequences were considered to be conserved miRNAs. Following the BLASTn search and further sequence analysis, 117 conserved miRNAs belonging to 33 families were found to be homologous to known miRNAs from other plant species, which had previously been deposited in the miRBase database. The number of members per miRNA family ranged between one and 15. The miR166, miR156 and miR157 families were the largest, with 15, 11 and 10 members, respectively, whereas 14 miRNA families had only a single member (Fig. 2A). Details of miRNA sequences and their reads are shown in Table S2. The expression of different members of the same family was found to be largely divergent. The abundance of miRNA families also varied drastically: miR157, miR166 and miR156 were most frequently represented in the library, with 154,908, 79,863 and 73,043 reads, whereas miR172, miR167, miR168 and miR396 were moderately abundant in the library with 6,411, 5,280, 4,373 and 2,500 copies. However, the expression of five miRNA families (miR828, miR858, miR4376, miR4495 and miR5083) showed the lowest abundance, with only one read (Fig. 2B).

**Figure 3:**
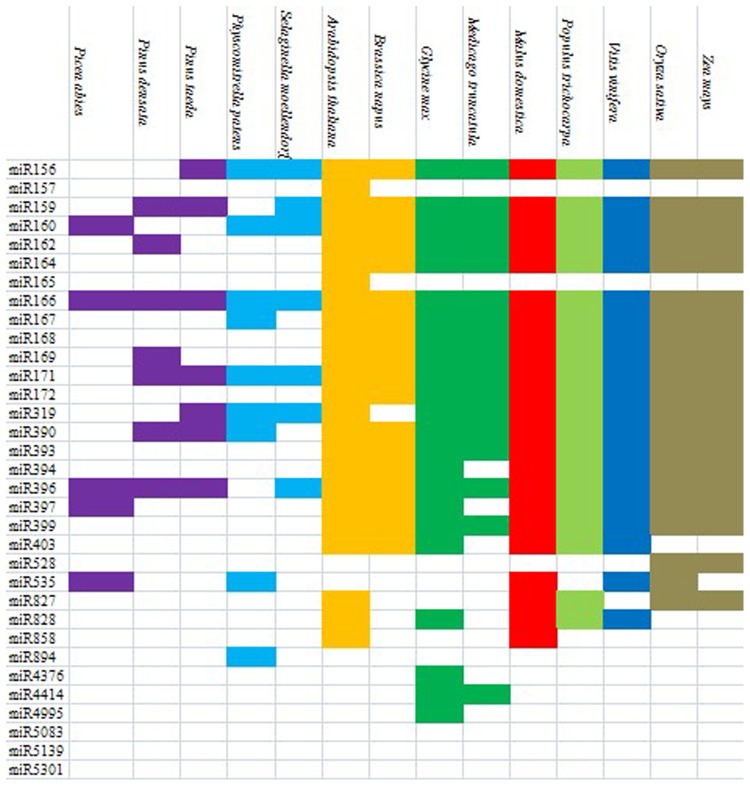
Conserved miRNA families from peach, designated as pmu on the first column, and their homologues in other plant species.

To investigate the evolutionary roles of these conserved miRNAs, extensive comparisons were performed against known miRNAs in 14 other plant species (Fig. 3). Among the miRNA families in peach, miR156, miR159, miR160, miR166, miR171, miR319, miR390 and miR396 showed a high conservation in plants, indicating that these 12 peach miRNA families are ancient. In addition, 11 miRNA families (miR162, miR164, miR167, miR168, miR169, miR172, miR393, miR394, miR397, miR399 and miR827) shared a high conservation in both dicotyledons and monocotyledons. However, 11 miRNA families were less conserved among the 14 plant species. Three families (miR5083, miR5139 and miR5301) had no corresponding homologues. The others (miR157, miR165, miR894, miR4376 and miR4995) had only one homologue among the 14 plant species. Furthermore, miR403 and miR828 was only found in dicots, whereas miR528 was only identified in monocots.

### Identification of Peach-specific miRNAs

**Figure 4:**
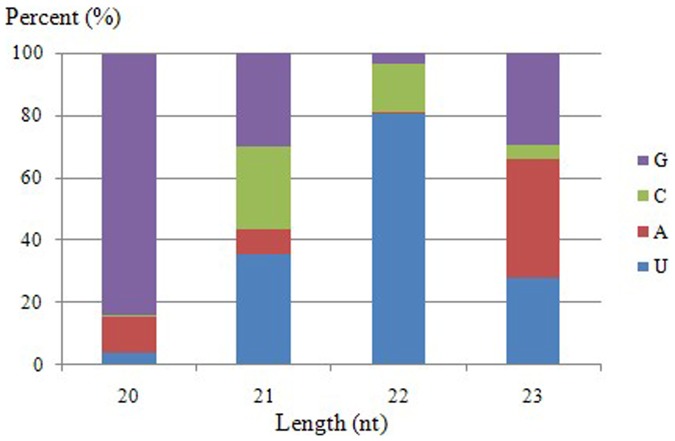
First nucleotide bias in novel miRNA candidates in peach.

The unannotated small RNAs of 3,690,979 unique reads matching the genome was subjected to secondary structure analysis. After using the criteria suggested by Meyers et al. [39] and the program MIREAP developed by BGI, 186 putative novel miRNAs were identified. The first nucleotide bias analysis showed that uracil was the most frequently used first nucleotide in miRNAs of peach, with 10,801 uracil nucleotides (41.68%). This result was agreement with previously reports that the uracil nucleotide is dominant at the first position of the 5′ end for the majority of miRNAs (Fig. 4). Our sequence analysis showed that the minimum folding free energies (MFEs) of potential novel miRNA precursors ranged from 18.33 to 171.23 kcal/mol with a mean of 53.13 kcal/mol using mFold software (Table S3). Among the miRNA candidates, 46 were identified with complementary miRNA*s. In contrast to the common or evolutionarily conserved miRNAs, the predicted novel miRNAs were often expressed at a very low level, 77.96% of which were sequenced fewer than 50 times. From 186 novel miRNAs candidates, only three miRNAs (miRC57, miRC162 and miRC167) were sequenced more than 1,000 times and 11 miRNAs more than 500 times.

### miRNA Validation and Quantification by RT-qPCR

**Figure 5:**
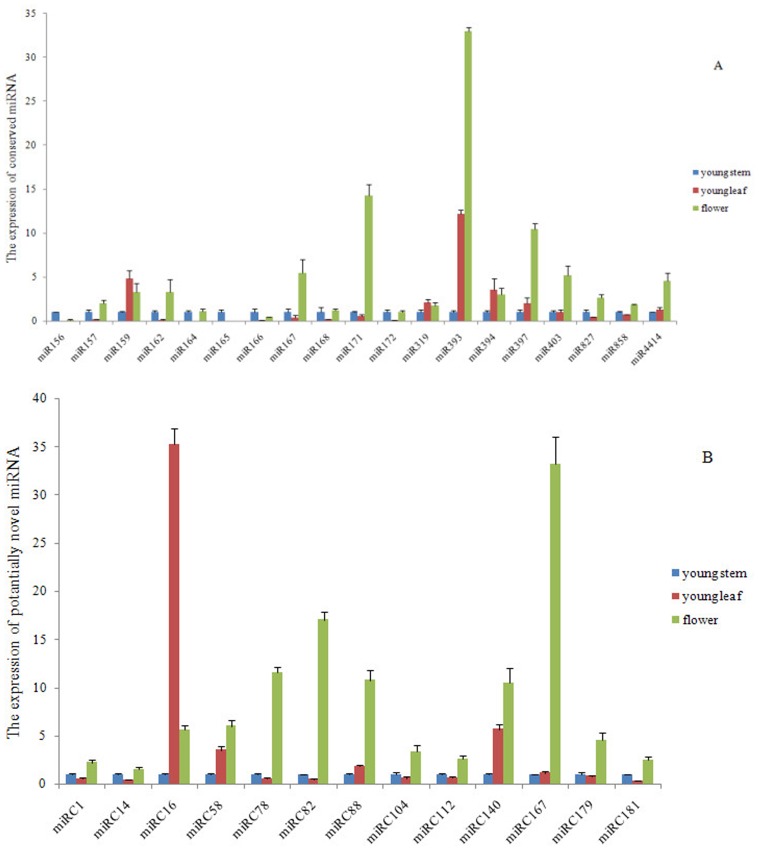
Relative expression levels of peach miRNAs in different tissues. A, Relative expression levels of conserved miRNAs; B, relative expression levels of novel miRNAs.

To evaluate the differential expression of the miRNAs in three tissues and to assess their potential roles in regulating the expression of the genes, the expression of 19 conserved miRNAs and 13 novel miRNA candidates was analysed using poly (A) qRT-PCR. These qRT-PCR analyses demonstrated that the expression of miRNAs differed greatly in young leaves, stems and flowers. The expression of 8 conserved (miR166, miR168, miR319, miR394, miR399, miR827, miR894 and miR5139) and six novel miRNAs (miRC1, miRC14, miRC16, miRC112, miRC179 and miRC181) showed no significant change in different tissues (Figs. 5A and 5B). It was notable that the majority of the tested miRNAs were more abundant in flowers than in young leaves and stems, which suggests that these miRNAs might play important roles in peach flower development, whereas miR393 and miRC16 mainly accumulated in young leaves.

### Summary of the Degradome Library Data

In higher plants, most miRNAs regulate their target genes via cleavage, which normally occurs at the tenth nucleotide of the complementary region between the miRNA and the mRNA [36]. Three degradome libraries, which capture the cleaved mRNAs were constructed from young leaves, stems and flowers in peach. After submitting libraries for parallel analysis of RNA end (PARE) Solexa sequencing [33], [40], a total of 12,295,775, 11,921,348, and 12,004,101 raw reads and 7,974,504, 8,329,611, 8,766,508 unique raw reads were obtained from peach leaf, stem and flower libraries, respectively (Table 2). In total, 5,320,695, 5,004,686 and 5,292,314 reads (referred to as cDNA mapped reads), which perfectly mapped to the peach cDNA database, remained. Finally, 22,058, 2,176 and 22,420 reads, which contained miRNA-mediated cleavage sites, were identified.

**Table 2:** Data summary of three degradome libraries of peach.

Type	Leaf library	Stemlibrary	Flowerlibrary
Raw Reads	12,295,775	11,921,348	12,004,101
Unique Raw Reads	7,974,504	8,329,611	8,766,508
cDNA Mapped Reads	5,320,695	5,004,686	5,292,314
Total Number of input cDNAs	34,809	28,701	28,701
Number of covered cDNAs	22,058	21,767	22,420

### Identification and Classification of Targets for known miRNAs and Novel miRNA Candidates

A total of 259 target mRNAs were identified from three degradome libraries in peach, 172 of which were the target genes for 26 conserved miRNA families and 87 of them for 38 putative novel miRNAs (Table S4). It should be noted that conserved miRNA families on average targeted more gene transcripts compared to novel miRNA candidates. The largest number of targets was shown by miR156, miR172 and miR396, with 25, 21 and 22, respectively. The target genes were classified into five categories (0–4), based on their number. It was shown that 29.30%, 25.90% and 55.43% targets belonged to category 0 from peach flowers, young stems and young leaves, respectively. Target gene categories 1 and 3 had fewer members (Table 3).

**Table 3:** The percentage of target gene categories in three tissues.

Category	Flower	Young stem	Young leaf
0	29.30%	25.90%	55.43%
1	5.38%	0.60%	0.37%
2	25.27%	30.92%	24.72%
3	1.61%	2.41%	0.75%
4	38.44%	40.16%	18.73%

Some of the target genes were abundantly represented as conserved miRNA targets, including many transcription factors such as *ARF*, *MYB*, *NF-Y*, *GROWTH REGULATORY FACTOR*, *HD-ZIP*, *PPR*, *SBP* and the NAD (P)-binding protein-encoding gene and genes encoding ribosomal proteins. In contrast, we found some other miRNA targets such as genes encoding mitochondrial acyl carrier protein, vacuolar ATPase assembly integral membrane protein, proline extensin-like receptor kinase, galactose oxidase/kelch repeat superfamily protein, ankyrin repeat family protein, cell differentiation protein and jasmonate-ZIM-domain protein (Table S4).

Among the 568 miRNA target genes, 197 were present in all three tissues; 123 and 70 targets were only found in peach stems and flowers, respectively, 102 targets were identified in stems and flowers, whereas 76 targets were found in leaves and stems (Table S5).

## Discussion

### High-throughput Sequencing of Peach Small RNAs

The study of miRNAs as regulators of gene expression has been extensively performed in plants in recent years [41]. The majority of miRNA genes in plants show high conservation and rapid evolution. Deep sequencing technologies allow miRNAs to be identified at an increased depth. Species-specific miRNAs, which should be greater than the number of conserved miRNAs [42] and only 47 peach-specific and 47 known miRNAs or families with distinct expression patterns were identified in peach by deep sequencing [28]; In this study, 117 conserved miRNAs and 186 novel miRNA candidates were identified. We found seven novel lowly-conserved miRNA families (miR528, miR4376, miR4414, miR4995, miR5083, miR5139 and miR5301) and 186 novel miRNA candidates.

### miRNA Expression Signatures Associated with Tissue Development

The differential accumulation of miRNAs in different tissues is common [35]. Notably, 12 from 19 conserved miRNAs and 12 from 13 novel miRNA candidates were highly expressed in flowers according to RT-qPCR data. It has been suggested that these miRNAs might play an important role in organ boundary formation or developmental processes. In addition, the expression of miRC16 was much higher in leaves than in flowers and stems, suggesting that it might be associated with leaf development.

### Identification of Previously Undiscovered miRNAs Targets

To identify previously undiscovered miRNAs target, three degradome libraries were constructed. The identification of miRNA targets in the libraries was low, regardless of whether they were conserved or novel. It was also noted that miRNAs with a single nucleotide difference within the same family make them functionally distinct because of their interaction with different AGO complexes [43]. Recently, miRNA sequence length variation was also reported to have dramatic effects on miRNA functions [43]–[45]. The targets of conserved and species-specific miRNAs appear to be involved in various processes in plants; many of which were previously uncharacterised. MiR156 and miR157 not only targeted *SQUAMOSA* promoter-binding protein-like (SBP domain) transcription factors, but also genes encoding proteins associated with energy metabolism, glucose metabolism, redox status and ion transport (Table S4). In this study, miR159 not only targeted *MYB* transcription factors but also regulated the expression of genes encoding ENTH/VHS family proteins, cytokinin oxidase/dehydrogenase, and transferases. In addition, *UDP-XYL* synthase was a target of miR164. Thus, the novel peach miRNAs targeted different genes with a wide variety of predicted functions.

## Conclusions

In this study, we constructed a small RNA library and three degradome libraries from three tissues for deep sequencing and identified 117 conserved miRNAs and 186 novel miRNA candidates. In addition, we identified 172 targets for conserved miRNA families and 87 for novel miRNAs, using recently developed tools for the global identification of miRNA targets. Peach represents a model for fruit trees in the *Rosaceae*. We expect to identify more peach-specific miRNAs and their targets and to obtain a better insight into the miRNA networks in peach.

## Materials and Methods

### Plant Materials

*Prunus persica* cv. Lovell peach trees were grown in the garden of Nanjing Agricultural University, China. Young emerging leaves, young stems and flower buds were collected from ‘Lovell’ peach trees before the end of flowering. After collection, all samples were immediately frozen in liquid nitrogen and stored at −70°C until use.

### Small RNA and Degradome Library Construction for Solexa Sequencing

A low molecular weight (LMW) RNA was isolated from mixed tissues (young leaves, young stems and flower buds) using the CTAB reagent according to the procedure previously described by Wang et al. [46]. The sample was sequenced by the Beijing Genomics Institute (BGI) (Shenzhen, Guangdong Province, China) using high-throughput pyrosequencing technology developed by Solexa. Three total RNAs were extracted from peach: young leaves, young stems and flowers. The degradome cDNA libraries were prepared following the procedures previously described by German et al. [40], [47].

The small RNA library and three degradome libraries sequencing data are available under NCBI-GEO accession no: GSE49579.

### Bioinformatic Analysis of Sequencing Data

After removing low quality reads and trimming adaptor sequences, the high-quality small RNA reads ranging from 18 to 30 nucleotides were obtained from the sRNA raw data. Small RNA sequences matching non-coding rRNA, tRNA, snRNA and snoRNA in the Rfam 10 (http://www.sanger.ac.uk/resources/databases/rfam.html) and NCBI Genbank databases were removed. The remaining sequences were searched against the miRBase database v18.0 (http://www.mirbase.org, release 18) with up to two mismatches, to identify conserved miRNAs. The sequences that did not map to any miRNAs in miRBase were analysed for predictions to identify novel miRNAs by the program MIREAP (developed by BGI) with default parameters for mapping the peach genome and obtaining all candidate precursors with hairpin-like structures of novel miRNA candidates [31]. Secondary structures of novel miRNAs were also checked using Mfold 3.2 [48].

The conserved miRNA families identified in peach were compared with known miRNA families in 14 other plant species, including *Picea abies*, *Pinus densata*, *P. taeda*, *Physcomitrella patens*, *Selaginella moellendorffii*, *Brassica napus*, *Glycine max*, *Medicago truncatula*, *Malus domestica*, *Populus trichocarpa*, *Vitis vinifera*, *Oryza sativa*, *Zea mays*. Peach miRNAs that had corresponding homologues in more than 10 plant species were classified as highly conserved miRNAs. While miRNAs that had corresponding homologues in at most 3 plant species were considered as less conserved miRNAs.

The construction of degradome libraries differed considerably from past efforts [33], [49] and followed the procedure of as Ma et al. [40], [47] with some modifications. (1) Approximately 150 ng of poly (A)^+^ RNA was used as input RNA and for annealing with biotinylated random primers; (2) Strapavidin capture of RNA fragments was performed via Biotinylated Random Primers; (3) 5′ adaptor ligation was only performed to those RNAs containing 5′-monophosphates; followed by (4) reverse transcription and PCR; (5) libraries were sequenced using the 5′ adapter only, resulting in the sequencing of the first 36 nucleotides of the inserts that represented the 5′ ends of the original RNAs. The purified cDNA library was used for cluster generation on Illumina’s Cluster Station and then sequenced on Illumina GAIIx following the vendor’s instructions for running the instrument. Raw sequencing reads were obtained using Illumina’s Pipeline v1.5 software following sequencing image analysis by the Pipeline Firecrest Module and base-calling by the Pipeline Bustard Module. A Public software package, CleaveLand 3.0 was used for analysing the generated sequencing data.

The peach genome sequences, CDS sequences and gene annotation information were obtained from GDR (www.rosaceae.org).

### miRNA Validation and Quantification by RT-qPCR

The LMW RNAs, independently extracted from peach leaves, young stems and flowers were used for RT-qPCR. The cDNA was mixed with 2× SYBR Green Mix (Takara, Japan), together with the miRNA-specific primers and a universal reverse primer (URP) in a final volume of 20 µL. Samples were analyzed in biological triplicate in a 96-well plate. All quantitative PCRs were performed in an ABI 7300 Real-Time PCR System (Bio-Rad) and the PCR conditions were 95°C for 30 s, 40 cycles of: 95°C for 30 s and 60°C for 34 s and a melting curve immediately after amplification. Each reaction was performed in triplicate. The relative miRNA expression was quantified using the comparative ΔΔCT method [50]. All expression profiles were normalised to expression levels in the stem. 5.8S rRNAs [51] was used as an internal control. The primer sequences are shown in Table S1. There three biological replications in every sample.

### Additional Information

Accession codes: all of raw sequencing data of this paper have been deposited at GEO under accession GSE49579. P. persica mixed tissues for small RNA sequencing data have been deposited at GEO under accession GSM1202204. *P. persica* degradome Sequencing data for P. persica are available in the GEO under accession GSM1202205, GSM1202206 and GSM1202207.

## Supporting Information

Table S1 Primer sequences of miRNAs.
(DOC)

Table S2 Peach homologs of conserved miRNAs.
(XLS)

Table S3 Detailed information of novel miRNA candidates in peach.
(XLS)

Table S4 The targets of known miRNA in peach.
(XLS)

Table S5 Expression of 568 target genes in different tissues.
(XLS)
