# Supplementary material for: Identification of miRNAs and Their Target Genes in Peach (Prunus persica L.) Using High-Throughput Sequencing and Degradome Analysis
Source: PLoS One. 2013 Nov 13;8(11):e79090. doi: 10.1371/journal.pone.0079090 (PMC3827290; doi:10.1371/journal.pone.0079090)
Supplement: Table S1 — Primer sequences of miRNAs. (DOC) [file pone.0079090.s001.doc]

Table S1 Primer sequences of miRNAs

| Name | Sequence (5’-3’) | Name | Sequence (5’-3’) |
| --- | --- | --- | --- |
| miR156 | CTGACAGAAGATAGAGAGCAC | miR157 | TTGACAGAAGATAGAGAGCAC |
| miR159 | TTTGGATTGAAGGGAGCTCTA | miR162 | TCGATAAACCTCTGCATCCAG |
| miR164 | TGGAGAAGCAGGGCACG | miR165 | TCGGACCAGGCTTCATC |
| miR166 | TCGGACCAGGCTTCATTC | miR167 | TGAAGCTGCCAGCATGATCT |
| miR168 | TCGCTTGGTGCAGGTCGG | miR171 | TGATTGAGCCGTGCCAATATC |
| miR172 | AGAATCTTGATGATGCTGCATAAAA | miR319 | TTGGACTGAAGGGAGCTCCCT |
| miR393 | TCCAAAGGGATCGCATTGATC | miR394 | TTGGCATTCTGTCCACCTCC |
| miR397 | TCATTGAGTGCAGCGTTGATG | miR403 | TTAGATTCACGCACAAACT |
| miR827 | TTAGATGACCATCAACAAACAAA | miR858 | GTTTCACGTCGGGTTCACCA |
| miR4414 | AGCTGCTGACTCGTTGGTTCA | miRC1 | AACGGCGTCGTTTTGGACCAG |
| miRC14 | GGGTGAGAGGTTGCCGGAAAGA | miRC16 | GGAATGGGAGGATTGGGAAA |
| miRC58 | GAATTGTAGAAAATTAGTGGGTT | miRC78 | ATGGCGTTGGATGTTCCGGTGTA |
| miRC82 | TAGCCAAGGATGACTTGCCTG | miRC88 | AGGGACTAATGATGCAACACAAC |
| miRC104 | TGAGCCAAGGATGACTTGCCA | miRC112 | TTGATCACTGTAGCCACGGATA |
| miRC140 | TCCGAATGTAGCCTAGATGCC | miRC167 | TGATTCTTGTTGACGTGATGTAAA |
| miRC179 | CACTGATAGAATTGGGGGCAC | miRC181 | AGTCATGATCTCTTGGACTACAA |
| Reverse | | CCAGTAGCGTATGATGAGCA | |
| 5.8s rRNA forward primer | | CTCGGCAACGGATATCTCG | |
| 5.8s rRNA reverse primer | | CTAATGGCTTGGGGCG | |
| URP | CCAGTAGCGTATGATGAGCACAGAGTCTGAGATCACTCGTAGCGAGG-d(T)_33_-V(A/C/G)N(A/C/G/T) | | |
